# Association between electronic cigarette use and tobacco cigarette smoking initiation in adolescents: a systematic review and meta-analysis

**DOI:** 10.1186/s12889-021-10935-1

**Published:** 2021-06-03

**Authors:** Doireann O’Brien, Jean Long, Joan Quigley, Caitriona Lee, Anne McCarthy, Paul Kavanagh

**Affiliations:** 1 Health Research Board, Grattan House, 67-72 Lower Mount Street, Dublin 2, D02 H638 Ireland; 2 Health Intelligence, Strategic Planning and Transformation, Health Service Executive, 4th Floor, Jervis House, Jervis Street, Dublin 1, D01 W596 Ireland

**Keywords:** Electronic cigarette use, Vaping, Tobacco cigarette initiation, Smoking, Adolescents

## Abstract

**Background:**

This systematic review of prospective longitudinal primary studies sought to determine whether electronic cigarette (e-cigarette) use by teenagers who had never smoked conventional tobacco cigarettes (tobacco cigarettes) at baseline was associated with subsequently commencing tobacco cigarette smoking.

**Methods:**

The review followed the principles of a systematic review and meta-analysis. A key word search identified peer-reviewed articles published between 1 January 2005 and 2 October 2019 from seven bibliographic databases and one search engine. Using pre-prepared inclusion/exclusion criteria two researchers independently screened abstracts, and subsequently, full text papers. Selected articles were quality assessed in duplicate. Data on study participants characteristics, exposure and outcome measures were recorded in an adapted Cochrane Data Extraction Form. Feasibility assessment was done to detect clinical heterogeneity and choose an approach to meta-analysis. Analysis comprised pairwise random effects meta-analyses, and sensitivity and subgroup analyses.

**Results:**

From the 6619 studies identified, 14 one-off primary studies in 21 articles were suitable for inclusion. The participants ages ranged from 13 to 19 years and comprised teenagers based in Europe and North America. Nine of the 14 one-off studies, with follow-up periods between 4 and 24 months, met the criteria for inclusion in a meta-analysis of the association between ever use of e-cigarettes and subsequent initiation of tobacco cigarette use. Based on primary study adjusted odds ratios, our meta-analysis calculated a 4.06 (95% confidence interval (CI): 3.00–5.48, I^2^ 68%, 9 primary studies) times higher odds of commencing tobacco cigarette smoking for teenagers who had ever used e-cigarettes at baseline, though the odds ratio were marginally lower (to 3.71 times odds, 95%CI: 2.83–4. 86, I^2^ 35%, 4 primary studies) when only the four high-quality studies were analysed.

**Conclusion:**

The systematic review found that e-cigarette use was associated with commencement of tobacco cigarette smoking among teenagers in Europe and North America, identifying an important health-related harm. Given the availability and usage of e-cigarettes, this study provides added support for urgent response by policymakers to stop their use by teenagers to decrease direct harms in this susceptible population group, as well as to conserve achievements in diminishing tobacco cigarette initiation.

**Supplementary Information:**

The online version contains supplementary material available at 10.1186/s12889-021-10935-1.

## Background

E-cigarettes were first sold into Europe in 2006 and into the United States of America (USA) in 2007 and as of 2017, 433 brands of e-cigarettes and 15,586 flavours had been documented [1]. E-cigarette usage has become more common since 2007. One paper based on Eurobarometer surveys found that 63 million (or 14.6%) people residing in European Union member countries aged 15 or older had ever used e-cigarettes up to 2017 (95% confidence interval [CI]: 59.9 million–66.2 million), and 7.6 million (95% CI: 6.5 million–8.9 million) or 1.8% were current and regular e-cigarette users in 2017 [2]. Current (27.0%) and former smokers (41.3%) were more likely to be regular e-cigarette users than never smokers (12.8%) [2]. In the USA, the self-reported use of e-cigarettes among adults in the month prior to the survey was 3.2% in 2018, and use in young adults aged 18–24 years was more than double at 7.6% in the same time period [3].

The 2011–2018 USA-based National Youth Tobacco Surveys reported that current e-cigarette use among high-school students, increased from 1.5% in 2011 to 20.8% in 2018 [4]. Surveys of 11–16-year-old children in the United Kingdom (UK) reported between 7 and 18% had ever used e-cigarettes, and between 67 and 92% of children who regularly smoked had ever used e-cigarettes in 2015–2016 [5]. The surveys’ highest and lowest prevalence estimates for ever use of e-cigarettes among 11–16-year-olds who regularly smoked tobacco cigarettes was between 4 and 10%, while the highest and lowest estimates for regular e-cigarette use among the same cohorts was between 0.1 and 0.5% [5]. The Health Behaviours in School Children survey in Ireland reported that 22% of 12–17-year-old school children had ever used e-cigarettes in 2018, and 9% of 12–17-year-olds had used e-cigarettes in the 30 days prior to the survey [6]. Perikleous et al. found that e-cigarette use in the USA and Europe was associated with older teenagers, male teenagers, tobacco cigarette users, peer influence, daily smoking, and heavier smoking [7].

The emergence of e-cigarettes is a disruptive change challenging tobacco control globally [8–10], and countries are adopting different approaches to public health policy [11]. While the balance of harms and benefits of e-cigarettes for established smokers continue to be debated [12], their toxicological profile and the impact of nicotine on the developing adolescent brain make their use among children and young people especially concerning [13, 14].

In addition to the direct harm from e-cigarettes, one of the main concerns for this vulnerable group is potential for e-cigarette use to lead to tobacco cigarette use with its associated lifelong harms, and thereby undermining tobacco control initiatives. This is a critical area where evidence is required to inform public health policy [15].

Four published systematic reviews investigated whether e-cigarette use to led to tobacco cigarette use. Soneji et al., combining seven primary studies in their synthesis, reported that e-cigarette use is associated with an increased risk of subsequent cigarette smoking initiation and current cigarette smoking in young people aged 14–30 years, even after adjusting for potential confounding by demographic, psychosocial, and behavioural risk factors [16]. Glasser et al. in a narrative analysis observed that e-cigarette use is associated with consequent smoking in young people [17]. Khouja et al. found convincing evidence of an association between e-cigarette use and subsequently cigarette smoking in their meta-analysis of baseline non-smokers aged up to 30 years (OR: 4.59, 95% CI: 3.60 to 5.85, I^2^ 88%) [18]. Aladeokin and Haighton, in a three-study meta-analysis, demonstrated that UK-based teenagers who use e-cigarettes were six times more likely to smoke tobacco cigarettes. Only one of the three systematic reviews focused exclusively on teenagers, and this review included UK-based teenagers only [19].

Given the significance of teenage years in establishing future smoking behaviour internationally [20], the aim of the systematic review was to build on existing work by determining if e-cigarette use by adolescents who never smoked tobacco cigarettes at baseline was associated with subsequent initiation of cigarette smoking through a meta-analysis of the longitudinal prospective studies. The final included studies represent a wider population from Europe and North America than in Aladeokin and Haighton [19]. Our review includes a larger number of studies and participants and so provide a more stable and generalisable estimate.

## Methods

This study adhered to the tenets of a systematic review and meta-analysis.

### Literature search strategy and inclusion-exclusion criteria

One author did a structured and robust search of seven databases and one search engine for peer-reviewed literature on e-cigarettes published between 1 January 2005 and 15 April 2019. The sources searched were Ovid MEDLINE (Additional file 1: Appendix 1), Cochrane Library, Ovid PsycINFO, Elsevier Embase, PROSPERO, LILACS, CORE.ac.uk, and Google Scholar. The searches were repeated twice, using Ovid MEDLINE, with a final date 2 October 2019*.* Our keywords were based on different English words for e-cigarette, for example, e-cig*, e-liquid, vape, vaping, cigalike, electronic nicotine delivery system (ENDS), and electronic non-nicotine delivery. Non-English words for these concepts were also used, for example, e-sigaret*, E-zigarette, and e-papieros. Using predefined inclusion/exclusion criteria (Table 1), two authors independently completed three rounds of document screening. We did backward citation searching using bibliographies of all papers included in the review. Our PRISMA diagram is presented in Additional file 1: Appendix 2. This systematic review observed the Centre for Reviews and Dissemination’s guidance for completing reviews in health care [21].

**Table 1:** Population, intervention, comparator and outcome inclusion criteria for review question

Element	Description
Population	Adolescents who never smoked tobacco cigarettes at baseline. The age of the included population was between 13 and 19 years at baseline. The could be living in any country around the Globe.
Exposure	Any e-cigarette vaping at baseline or in the past
Comparators	Non-electronic cigarette user
Outcomes	Initiation of tobacco cigarette smoking at follow-up
Study design	Longitudinal cohort studies
Search dates	2005–2019

### Quality assessment and data extraction

Two authors independently evaluated the quality of the 14 studies using the National Heart, Lung, and Blood Institute’s (NHLBI’s) 14-item quality assessment tool for observational studies (Additional file 1: Appendix 3) [22]. One author extracted key population, exposure, and outcome data from the included papers into an adapted Cochrane Data Extraction Form [23] and these extracted data were verified by another author.

### Statistical analyses

A meta-analysis feasibility assessment including the 14 studies was done to decide whether to do meta-analysis and to choose the most suitable meta-analysis method [24–26]. The feasibility analysis assessed the studies’ similarities and differences in outcome, exposure, unit of measurement, and length of time to follow-up. A pairwise random effects meta-analyses using the longitudinal cohort studies adjusted odds ratios to evaluate outcomes of studies exposures was completed for the outcome ‘initiated tobacco cigarette smoking’ for the studies’ follow-up periods [27–29]. The *I*^*2*^ inconsistency index describes the percentage of the variability in treatment effects that is due to statistical heterogeneity rather than sampling error (chance). We performed two sensitivity analyses: one including studies rated as high-quality and another including studies that controlled for three domains of confounding (demographic, interpersonal, intrapersonal). Subgroup analyses were also completed by year of data collection (baseline data collection pre 2014 compared to baseline data collection 2014 to date), geographical region (North America versus Europe) and length of follow-up (less than 12 months compared to 12 months or more). A level of evidence [30] and a GRADE recommendation [31] were ascribed to the main outcome. The methods for this paper are described in detail elsewhere [32].

## Results

### Study and population characteristics

The searches retrieved 6619 studies (6510 papers from the initial searches, plus 109 papers from supplemental searches). Two researchers selected 21 papers for inclusion in the study (Additional file 1: Appendix 2); comprising 14 unique longitudinal prospective studies (Additional file 1: Appendices 4–8). The data in the primary studies were collected between 2013 and 2016 and their longitudinal follow-up periods ranged from 4 months to 2.5 years. Only one study had two follow-up time points [33]. Fifteen primary papers were completed using North American populations [33–47] and six primary papers were based on European populations [48–53]. The studies’ populations ages ranged from 13 to 19 years at baseline. The included studies had a range of research questions related to e-cigarette use; 17 enquired about ever use of e-cigarettes [33, 36–40, 42, 44–53], and 4 queried current e-cigarette use in the past 30 days [35, 40, 43, 44]. Data on e-cigarette type, generation or liquid were not asked about in any study. The papers assessed tobacco cigarette smoking during follow-up as an outcome variable: 18 papers studied ever use of tobacco cigarettes between baseline and follow-up [33, 35, 37–52], and 4 queried past 30-day use of tobacco cigarettes [39, 40, 42, 44]. The publications completing regression analysis included potential confounding variables as covariates in their regression model, ranging from the inclusion of 3 variables to the inclusion of 17. Based on previous research, we grouped the covariates into three groups: demographic (e.g. age, gender, ethnicity, family affluence), interpersonal (e.g. number of friends/family members that smoke) or intrapersonal (e.g. such impulsivity, sensation seeking, rebellion) [17]. One paper [36] collected data on variables from a single domain, while eight papers [35, 38, 43, 46, 47, 50, 51, 53] had variables representing two domains, and ten papers [33, 34, 39, 40, 42, 44, 45, 48, 49, 52] had variables from all three domains.

### Quality assessment

Overall, using the NHLBI quality assessment tool [22], we considered four studies to be high-quality [39, 44, 47, 52] as they had a representative and clearly defined sample with a participation rate of more than 50%, a loss to follow-up rate of 20% or less, and a sample size justification or variance calculation for the main outcomes (Additional file 1: Appendix 3). The remaining studies in the meta-analysis were judged to be moderate quality and tended to have higher loss to follow up or lower participation rates.

### Feasibility assessment

**Fig. 1:**
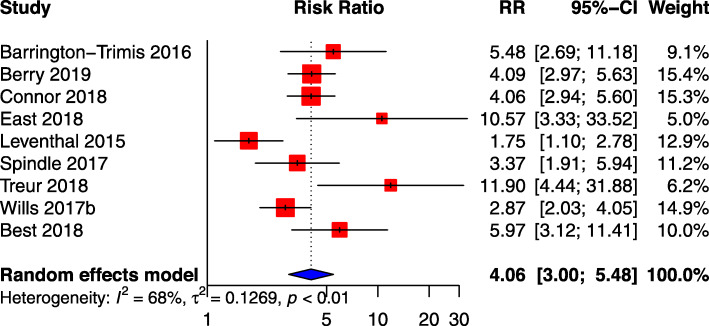
Meta-analysis results, using primary study adjusted odds ratio (AOR), for association between ever e-cigarette use and subsequent smoking

In order to ascertain whether a meta-analysis was appropriate and which studies should be included, a feasibility analysis was conducted, assessing if the primary study authors employed the same method of analysis considering outcome, exposure, unit of measurement, and length of time to follow-up (Additional file 1: Appendix 9). Based on these criteria, nine studies with 16,808 participants were considered eligible for meta-analysis of ever using e-cigarette at baseline and smoked tobacco cigarettes at any time during the follow-up period (Fig. 1), while three studies of past-30-day e-cigarette use at baseline and smoked tobacco cigarettes at any time during the follow-up period were eligible.

### Ever e-cigarette use at baseline and subsequent cigarette smoking at follow-up

Our ever e-cigarette-use meta-analysis was based on the primary study adjusted odds ratios and calculated a combined 4.06 (95%CI: 3.00–5.48, I^2^ 68%) times higher odds of initiating smoking tobacco cigarettes for those who had ever used e-cigarettes at baseline, although this combined odds ratio decreased marginally to 3.71 times (95%CI: 2.83–4. 86, I^2^ 35%) when only the four high-quality studies [39, 44, 47, 52] were analysed in a sensitivity analysis (Additional file 1: Appendix 10). The initial meta-analysis model had high statistical heterogeneity, sensitivity analysis restricted to high-quality studies had moderate statistical heterogeneity.

One additional sensitivity analysis was completed on six studies [33, 39, 44, 48, 49, 52] that controlled for the three domains of covariates – that is, demographic, interpersonal, and intrapersonal factors. The results of this sensitivity analysis (OR: 3.82; 95% CI: 2.66–5.48; I^2^ 69%) were like the results for the high-quality studies sensitivity analysis, but the level of heterogeneity remained high (Additional file 1: Appendix 10).

Three subgroup analysis were done (Additional file 1: Appendix 10). The first compared studies which collected data pre-2014 [33, 39, 47] with those which collected their initial data from 2014 onwards [38, 44, 48, 49, 51, 52], due to the rise in e-cigarette use that was noticed around this time [54]. The combined OR for studies which collected data from 2014 onwards increased substantially (pre-2014 AOR: 2.81, 95%CI: 2.45–3.72 I^2^ 78%); compared to 2014 onwards (5.16, 95%CI: 3.69–7.21 I^2^ 38%). However, the confidence intervals overlap indicating that they are not statistically significantly different.

The second subgroup analysis compared the length of time to follow-up, as studies included in the analyses had follow-up periods which ranged from 4 months to 2 years. However, as only two studies had follow-up periods of less than 1 year [49, 51] including one study which had a very small sample size, the meta-analysis for this subgroup did not provide useable results.

Finally, considering the significance of the geographical, regulatory, and cultural context of these studies, we compared European studies with those from the USA. The combined OR was higher in the European studies (OR: 6.22, 95%CI: 3.73–10.38 I^2^ 54%) [48, 49, 51, 52] compared with the USA studies (OR: 3.18, 95%CI: 2.26–4.47 I^2^ 65%) [33, 38, 39, 44, 47]. However, the confidence intervals overlap indicating that they are not statistically significantly different.

### Past-30-day e-cigarette use at baseline and subsequent cigarette smoking at follow-up

**Fig. 2:**
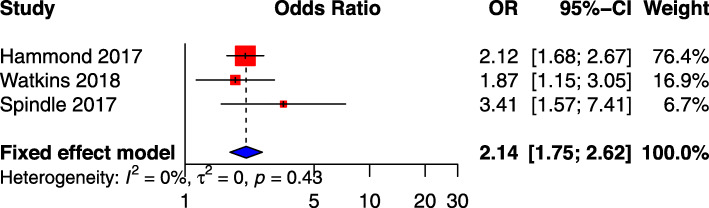
Meta-analysis results, using primary study adjusted odds ratio (AOR), for association between past 30-day e-cigarette use and subsequent smoking

Four studies measured the effect of past-30-day e-cigarette use at baseline and subsequent cigarette smoking at follow-up [35, 40, 43, 44]. One of the four studies was excluded following feasibility analysis prior to meta-analysis. A meta-analysis was completed using the remaining three primary studies adjusted odds ratios (Additional file 1: Appendix 10) and included 30,018 participants. The meta-analysis identified a significant positive association between past-30-day e-cigarette use at baseline and subsequent cigarette smoking initiation at follow-up (OR: 2.14; 95% CI: 1.75–2.62; I^2^ 0%) (Fig. 2) [35, 40, 44].

### Level and certainty of evidence

We ascribed a level of evidence of 3 using British Medical Journal guidelines [30], as this is a systematic review of cohort studies, some of which had high loss to follow-up and/or very small sample sizes. With respect to certainty of evidence [31] and taking account of study design and statistical heterogeneity, we have moderate confidence that the true effect is likely to be close to the estimated effect for starting smoking at follow-up for those who had ever used e-cigarettes at baseline.

## Discussion

### Main findings

We calculated a more precise and generalisable estimate of the odds of tobacco cigarette smoking initiation following ever use of e-cigarettes in adolescents in Europe and North America. We identify a four-fold increased likelihood between e-cigarettes use and initiating smoking tobacco cigarettes in adolescents in a combined analysis of nine cohort studies conducted with follow-up periods between 4 and 24 months. Sensitivity and subgroup analysis support the relationship between ever using e-cigarettes and initiating smoking tobacco cigarettes. Six studies controlled for confounding under three domains (demographic, interpersonal, and intrapersonal) while examining the association between using e-cigarettes and initiating smoking tobacco cigarettes, and had a similar, significant estimate of effect in meta-analysis. The four high-quality studies also had a similar estimate of effect but lower statistical heterogeneity. The remainder of the studies were judged to be moderate quality because of their small sample sizes and/or high loss to follow-up.

The longitudinal prospective study design used to evaluate the relationship between e-cigarette use and initiation of tobacco cigarette smoking permits researchers to start the process of establishing a causal relationship. Across all primary studies included in the meta-analysis, the adjusted odds ratios and confidence intervals are consistent, and the strength of association is statistically significant. Moreover, the use of e-cigarettes which occurred before initiating smoking, fulfils the criteria for a temporal relationship. Finally, two studies in this review have illustrated a dose-response relationship. We have moderate confidence that the true effect is probably close to the estimated effect for initiating smoking at follow-up for those who had ever used e-cigarettes at baseline, as all meta-analyses indicate that there is a significant positive association between using e-cigarettes at baseline and smoking tobacco cigarettes at follow-up, and this effect size is quite large; the findings are statistically significant, consistent, and the exposure occurred before the outcome. We also found a significant two-fold positive association between past-30-day e-cigarette use at baseline and subsequent cigarette smoking initiation at follow-up. This is a more restricted measure of exposure with a shorter duration during which the non-user can take up vaping, but shows the same positive association among smoking initiators demonstrating that e-cigarette vaping in non-smokers in the 30 days prior to data collection increases the odds of taking up tobacco cigarette smoking.

### Strengths and limitations

Given that there were 433 brands of e-cigarettes and 15,586 flavours documented by 2017 [1], the primary limitation of the research in this area is the heterogeneity of the exposure (e-cigarette) in terms of generation, product type, e-liquid and its nicotine content. The included studies did not ask specific questions about the e-cigarette used, and only two [51, 53] of the 21 studies measured the differences between nicotine and non-nicotine e-cigarettes. This is relevant as e-cigarettes industry is developing new products rapidly.

None of the cohort studies did biochemical verification of outcomes as they relied on the tried and tested questions about ever use, recent or last year use, and current or last 30 days use and these measures are accepted the world over for surveying the use of tobacco products, licit drugs, and illicit drugs [55]. The most common measure of both e-cigarette and cigarette use was ‘ever use’ of either product, an indicator which has been critiqued by researchers [56], as it did not observe whether the teenagers used the product once in their young life, or if they used it regularly. ‘Past-30-day use’ has gotten the same censure. However, the use of these indicators has been justified, with a recent study by Birge et al. finding that over two-thirds of smokers who ever consumed a single puff of a tobacco cigarette during adolescence became, for a time, regular smokers [57].

### Comparison with previous systematic reviews

The results of this up-to-date and comprehensive systematic review are in line with three published meta-analyses [16, 18, 19], that also found an association between initiation of e-cigarette use and subsequent smoking. However, this systematic review strengthens the evidence base for public health policy because it used nine studies concentrating on adolescents who resided in a wider geographical region, and the analysis also took account of the quality of the systematic reviews and control for confounding in the primary research. The World Health Organization, based on the U.S. National Academies of Sciences, Engineering, and Medicine’s systematic narrative review, reported that there is moderate evidence that young never smokers who experiment with e-cigarettes are at least two times more likely to experiment with smoking later, which is lower than, but in line with our findings [58]. The meta-analyses presented in this paper, however, includes newer studies not analysed in the U.S. Academies of Sciences review.

### Future research

An important question still to be answered relates to the principal catalysts in the relationship between e-cigarette and tobacco cigarette use. Researchers have tried to clarify the move from using e-cigarettes to smoking tobacco cigarettes through three theories which are the gateway theory [59], the common liability theory [60–63], and the catalyst model [64]. Future research is required to test these three theories (or elements thereof) in more depth.

In terms of the most appropriate study design for assessing causality, Etter recommended large longitudinal epidemiological studies which measure smoking onset, control for confounders, and include a propensity score measure of liability to smoking [56]. In addition to this, we propose exploring the phenomenon using explorative and in-depth quantitative and qualitative methods to understand the thinking and behaviour of adolescents who use e-cigarettes and subsequently move to tobacco cigarettes or use both, so as to provide enhanced interventions to prevent these practices.

Most research has taken place in Europe and North America: there is a need for research for cross-country research including low- and middle-income countries where the burden of tobacco cigarette use is the highest.

No study provided information on e-cigarette type, generation or liquid. It is important to note that e-cigarettes and their e-liquids were not a standard commodity or exposure but an umbrella term for a device that delivers nicotine and other products including flavourings [65]. Independent longitudinal research into e-cigarette devices and their liquids over several years is essential to identify and limit their long-term effects on human health.

### Implications for policymakers

Given over six million deaths attributable to smoking worldwide each year [66], tackling tobacco use continues to be a global health priority, with countries at different stages in controlling the epidemic and some high-income countries signalling intent to transition from tobacco control to tobacco endgame [67, 68]. E-cigarettes are a disruptive innovation raising new questions for health policymakers [8–10]. Debate on the harms and potential benefits of e-cigarettes has dominated tobacco control discourse [69], and became even more charged with the emergence of e-cigarette, or vaping, product use–associated lung injury (EVALI) in 2019 [70]. Two viewpoints, which often appear conflicting emerge: a harm minimisation approach is proposed to leverage a potentially favourable balance of harms and benefits for people who smoke so as to mitigate the overwhelmingly bleak odds they face from their use of combustible tobacco products [71], while a precautionary approach is advised given the many unknowns regarding e-cigarette use, evidence of tobacco industry interference, and previous false dawns of ‘safer’ tobacco products [72].

To find a way forward through these competing viewpoints, policymakers must carefully appraise evidence on risk, benefits, and trade-offs while understanding the framing and wider context of the debate [15, 73–75]. The U.S. National Academies of Sciences, Engineering, and Medicine, were commissioned by the Food and Drugs Administration to systematically review scientific evidence to inform e-cigarette policy [76], while in Europe the Scientific Committee on Health, Environmental and Emerging Risks has been mandated to assist the European Commission in assessing the most recent scientific and technical information on e-cigarettes as part of its review of the Tobacco Products Directive 2014/40/EU [77]. Similarly, the study presented in this paper was conducted as part of broader programme of evidence reviews to inform and support public health policy in Ireland, which included mapping of the harms and benefits of e-cigarettes (and their e-liquids) [65] and a systematic review of e-cigarettes role in smoking cessation [78]. We found that e-cigarettes (and their e-liquids) lead to acute harms such as poisoning, lung injury, and burns and blast injuries, a finding aligned with six other systematic reviews [58, 76, 79–82], and highlighted a need for continuing study using robust methods to measure the long-term health impacts of their use as these are not yet known. We also found that approved and regulated nicotine replacement therapies with established safety profiles were as effective as e-cigarettes in helping smokers quit [78].

Against a backdrop of often clashing harm reduction and precautionary viewpoints on e-cigarettes, mobilising evidence, while necessary, is difficult for policymakers This difficulty is exacerbated when the relationship between evidence and policy is seen as a linear “know-do-gap”, instead of recognising the “muddling through” of the policy process [83]. The harms, benefits and trade-offs to be considered by policymakers in the area of e-cigarettes are likely to be different across population groups and require a finely balanced blend of policies which are precautionary for vulnerable groups while retaining potential prospect of harm reduction for some highest-risk groups not amenable to other risk management measures.

In the case of any non-smokers, be they children, young people, or adults, e-cigarettes offer no benefits and present potential for harm from nicotine dependence and exposure to known toxins. The mandate for policy action to maximise protection of children and adolescents is further strengthened by the systematic review presented in this paper, in which we found that e-cigarettes were associated with initiation of tobacco cigarette smoking among adolescents. This is a identifying an important public health harm which undermines hard-won progress in tobacco control, that have been largely delivered through preventing smoking initiation in youth. Other researchers have noted that two-thirds of these adolescents may go on to smoke tobacco cigarettes for a period [57]. Children and adolescents should be offered the same protection from e-cigarettes as conventional tobacco cigarettes through a well-enforced regulatory regime of measures including age restriction on purchase, control of availability through licensing outlets, limits to product visibility and attractiveness, and appropriate pricing through taxation. Before exploring the potential for harm reduction for highest-risk groups and those for whom regulated pharmaceutical interventions do not work, policymakers should assure protection of children, adolescents and never smokers as their next evidence-informed, precautionary step through this complex and challenging policy process, so as to reduce direct harms from e-cigarettes in these vulnerable populations and to protect gains in reducing tobacco use initiation.

## Conclusion

The systematic review and meta-analysis found that e-cigarette use was associated with initiation of tobacco cigarette smoking among teenagers in Europe and North America, identifying an important public health harm. The meta-analysis presents a more precise and generalisable estimate of the odds of tobacco cigarette smoking initiation following ever use of e-cigarettes in teenagers. Given the widespread availability and use of e-cigarettes, this study further supports urgent action by policymakers to prevent their use by adolescents to reduce direct harms in this vulnerable population group as well as to protect gains in reducing tobacco cigarette initiation.

## Supplementary Information

**Additional file 1: **We present all additional information in one excel file (supplementary excel file) and within the file each tab is a single appendix. **Appendix 1.** (or Table 1) presents the Medline literature search strategy. **Appendix 2.** presents the study PRISMA flow chart. **Appendix 3.** presents the quality assessment tool and results. **Appendix 4.** presents characteristics of included studies. **Appendix 5.** presents primary research AOR for ever e-cigarette and cigarette use among adolescents in the selected longitudinal cohort studies. **Appendix 6.** presents primary studies ever use of e-cigarettes and cigarettes among adolescents in the selected longitudinal cohort studies using statistical measures such as adjusted relative risk, estimates, or standardised coefficient. **Appendix 7.** presents AORs for different frequencies of e-cigarette and cigarette use among adolescents in the selected longitudinal cohort studies. **Appendix 8.** presents adjusted relative risk for different frequencies of e-cigarette and cigarette use. **Appendix 9.** presents the feasibility assessment for meta-analysis and **Appendix 10.** presents the results of the meta-analysis, sensitivity analysis, and subgroup analysis.

## Abbreviations

AOR: Adjusted odds ratio
CI: Confidence interval
e-cigarette: electronic cigarette
e-liquid: electronic liquid for e-cigarette
EVALI: e-Cigarette, or vaping, product use–associated lung injury
NHLBI: National Heart, Lung, and Blood Institute
OR: Odds ratio
UK: United Kingdom
USA: United States of America
